# 2D Carbonaceous Materials for Molecular Transport and Functional Interfaces: Simulations and Insights

**DOI:** 10.1021/acs.accounts.4c00398

**Published:** 2024-08-27

**Authors:** Yujing Tong, Sheng Dai, De-en Jiang

**Affiliations:** † Department of Chemical and Biomolecular Engineering, Vanderbilt University, Nashville, Tennessee 37235, United States; ‡ Chemical Sciences Division, Oak Ridge National Laboratory, Oak Ridge, Tennessee 37831, United States; § Department of Chemistry, The University of Tennessee, Knoxville, Tennessee 37996, United States

## Abstract

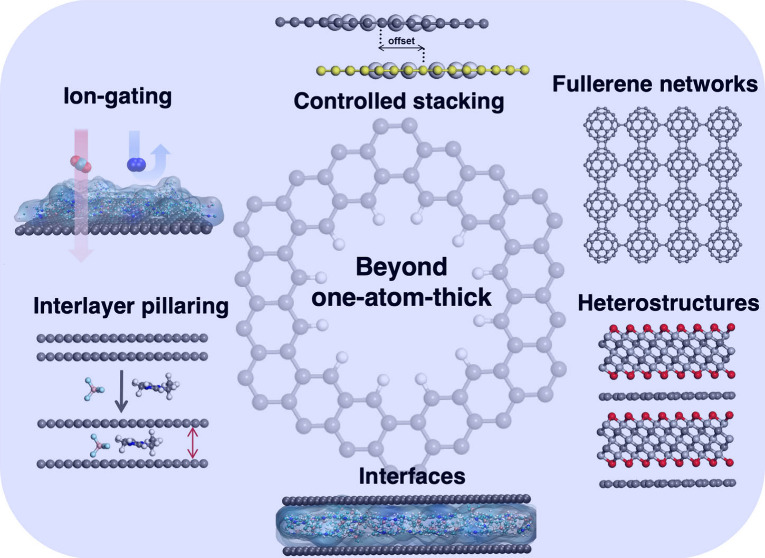

Carbon-based two-dimensional (2D) functional materials exhibit potential across a wide spectrum of applications from chemical separations to catalysis and energy storage and conversion. In this Account, we focus on recent advances in the manipulation of 2D carbonaceous materials and their composites through computational design and simulations to address how the precise control over material structure at the atomic level correlates with enhanced functional properties such as gas permeation, selectivity, membrane transport, and charge storage. We highlight several key concepts in the computational design and tuning of 2D structures, such as controlled stacking, ion gating, interlayer pillaring, and heterostructure charge transfer.

The process of creating and adjusting pores within graphene sheets is vital for effective molecular separation. Simulations show the power of controlling the offset distance between layers of porous graphene in precisely regulating the pore size to enhance gas separation and entropic selectivity. This strategy of controlled stacking extends beyond graphene to include covalent organic frameworks (COFs) such as covalent triazine frameworks (CTFs). Experimental assembly of the layers has been achieved through electrostatic interactions, thermal transformation, and control of side chain interactions.

Graphene can interface with ionic liquids in various forms to enhance its functionality. A computational proof-of-concept showcases an ion-gating concept in which the interaction of anions with the pores in graphene allows the anions to dynamically gate the pores for selective gas transport. Realization of the concept has been achieved in both porous graphene and carbon molecular sieve membranes. Ionic liquids can also intercalate between graphene layers to form interlayer pillaring structures, opening the slit space. Grand canonical Monte Carlo simulations show that these structures can be used for efficient gas capture and separation. Experiments have demonstrated that the interlayer space can be tuned by the density of the pillars and that, when fully filled with ionic liquids and forming a confined interface structure, the graphene oxide membrane achieves much higher selectivity for gas separations. Moreover, graphene can interface with other 2D materials to form heterostructures where interfacial charge transfers take place and impact the function. Both ion transport and charge storage are influenced by both the local electric field and chemical interactions.

Fullerene can be used as a building block and covalently linked together to construct a new type of 2D carbon material beyond a one-atom-thin layer that also has long-range-ordered subnanometer pores. The interstitial sites among fullerenes form funnel-shaped pores of 2.0–3.3 Å depending on the crystalline phase. The quasi-tetragonal phases are shown by molecular dynamics simulations to be efficient for H_2_ separation. In addition, defects such as fullerene vacancies can be introduced to create larger pores for the separation of organic solvents.

In conclusion, the key to imputing functions to 2D carbonaceous materials is to create new interactions and interfaces and to go beyond a single-atom layer. First-principles and molecular simulations can further guide the discovery of new 2D carbonaceous materials and interfaces and provide atomistic insights into their functions.

## Key References

- TianZ.; MahurinS. M.; DaiS.; JiangD. E.Ion-Gated Gas Separation Through Porous Graphene. Nano Lett.2017, 17, 1802–1807.28231000 10.1021/acs.nanolett.6b05121(1) The concept of ion-gated selective gas separation was proposed and demonstrated by molecular dynamics simulations, whereby the gas permeation through a graphene nanopore is gated by an anion from an ionic-liquid layer due to the favorable anion-pore interaction.
- WangS.; DaiS.; JiangD. E.Continuously Tunable Pore Size for Gas Separation via a Bilayer Nanoporous Graphene Membrane. ACS Appl. Nano Mater.2019, 2, 379–384.(2) A bilayer design was proposed to continuously tune the effective pore size at sub-0.1 Å resolution by varying the offset between two porous graphene layers, which was confirmed by molecular dynamics simulations to achieve highly selective separations of CO_2_, N_2_, and CH_4_.
- WangS.; MahurinS. M.; DaiS.; JiangD. E.Design of Graphene/Ionic Liquid Composites for Carbon Capture. ACS Appl. Mater. Int.2021, 13, 17511–17516.10.1021/acsami.1c0124233832221(3) Density functional theory and grand canonical Monte Carlo simulations were used to demonstrate a design in which an ionic liquid intercalates in between graphene layers as pillars to open up the gallery space in the ultramicropore range for carbon capture.
- TongY.; LiuH.; DaiS.; JiangD. E.Monolayer Fullerene Membranes for Hydrogen Separation. Nano Lett.2023, 23, 7470–7476.37540493 10.1021/acs.nanolett.3c01946(4) Nonequilibrium molecular dynamics simulations show that the square-latticed monolayer fullerene membrane based on the experimental quasi-tetragonal phase of the fullerene 2D network possesses the perfect pore size, shape, and geometry for H_2_/CO_2_ and H_2_/O_2_ separations.

## 1 Introduction

The discoveries of fullerenes (0D) in 1985 by Smalley et al.^5^ and carbon nanotubes (1D) in 1991 by Ijima^6^ broadened the scope of carbon allotropes. In 2004, scientists first isolated a graphene monolayer,^7^ spurring the development and extensive research of 2D materials. In 2022, monolayer fullerene network was isolated for the first time.^8^ 2D carbonaceous materials have made significant impacts across various fields, including separation,^9,10^ energy storage,^11^ catalysis,^12^ sensors,^13^ and superconducting materials.^14^

**Figure 1:**
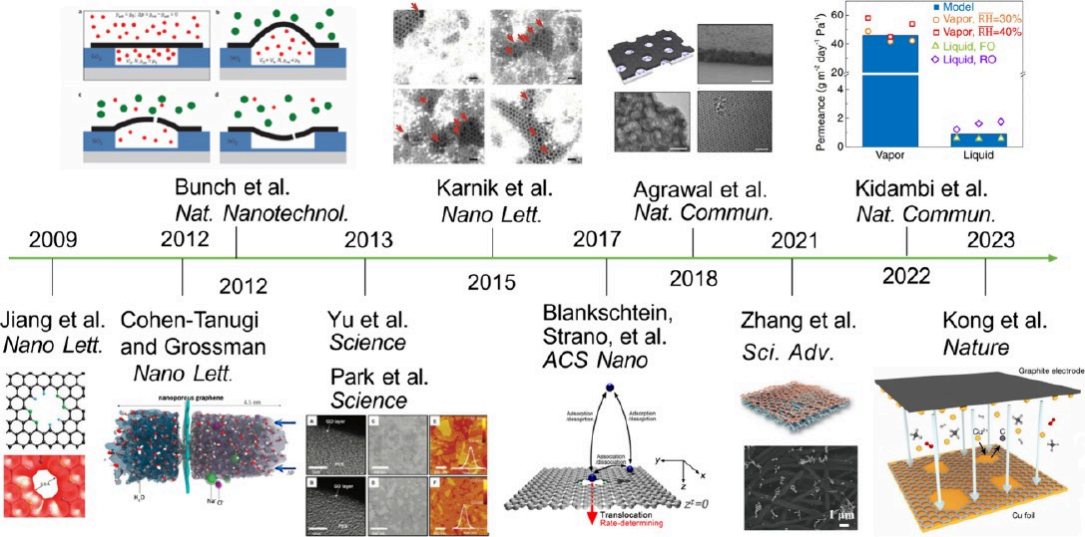
Timeline of porous graphene research for molecular separation and transport. Along the timeline, adapted with permissions from ref (15) (Copyright 2009 American Chemical Society), ref (16) (Copyright 2012 American Chemical Society), ref (17) (Copyright 2012 Springer Nature Limited), refs (18) and (19) (Copyright 2013 The American Association for the Advancement of Science), ref (20) (Copyright 2015 American Chemical Society), ref (21) (Copyright 2017 American Chemical Society), ref (22) (Copyright 2018 Agrawal et al.), ref (23) (Copyright 2021 The American Association for the Advancement of Science), ref (24) (Copyright 2022 Kidambi et al.), and ref (25) (Copyright 2023 Kong et al. under exclusive license to Springer Nature Limited).

The importance of the atom-thin nature of graphene in technology was recognized by Smalley in his 1996 Nobel lecture. One straightforward application of graphene is in membrane separations, as the permeance of a membrane is inversely proportional to its thickness. In 2009, Jiang et al.^15^ demonstrated a computational proof-of-concept of porous graphene for gas separation via size sieving using first principles molecular dynamics (FPMD) simulations and heralded the potential of introducing subnanometer pores in the graphene sheet for chemical separations by molecular sieving (Figure 1). In 2012, Cohen-Tanugi and Grossman^16^ used classical MD (CMD) simulations to demonstrate the potential of porous graphene for desalination. Meanwhile, Bunch and co-workers^17^ successfully fabricated single-layer graphene on a patterned Si wafer with cavities and measured gas leak rates after creating subnanometer pores with UV/ozone etching, experimentally demonstrating molecular sieving through porous graphene. In 2013, Park and workers^18^ as well as Yu and co-workers^19^ fabricated ultrathin graphene membranes for gas separation using reduced graphene oxides, thereby making the synthesis of graphene membranes more processable. In 2015, Karnik and co-workers^20^ reported nanoporous monolayer graphene for nanofiltration. In 2017, modeling and simulations by Blankschtein, Strano, and co-workers^21^ further elucidated the gas transport mechanisms through these membranes. In 2018, Agrawal and co-workers^22^ achieved a large area, crack-free, suspended graphene film by nanoporous-carbon-assisted transfer technique. In 2021, Zhang and co-workers^23^ developed nanofiber-supported monolayer graphene membranes for organic solvent nanofiltration. In 2022, Kidambi and co-workers^24^ found that water vapor permeates much faster than liquid water through monolayer porous graphene membranes. In 2023, Kong and co-workers^25^ developed a cascaded compression approach that allows for independent control of the density, mean diameter, standard deviation, and skewness of the pore size distribution in the creation of nanoporous graphene.

The field of porous graphene membranes for chemical separations as highlighted in Figure 1 demonstrates the power of simulation-guided discovery and development of functional 2D materials. There are more potential opportunities in exploring the interfaces and composites of graphene with other materials as well as other types of 2D materials such as covalently linked 2D fullerene monolayers. In this Account, we highlight some recent developments in simulation-guided understanding, design, synthesis, and testing of advanced 2D carbonaceous materials for molecular separations and functional interfaces from a few approaches: (i) stacking 2D layers; (ii) gating pores in 2D materials with ions; (iii) adding pillars between 2D layers; (iv) heterostructuring 2D layers of different types; (v) utilizing interstitial sites in covalently linked 2D fullerenes.

## 2 Layer Offset and Controlled Stacking

Controlled stacking of 2D porous layers enables the fine-tuning of the material’s properties by manipulating the stacking order^26^ and pore alignment.^27^ It involves the deliberate arrangement of layers with inherent porous structures in a specific sequence or orientation. The extent of the offset and the specific stacking order could be thoroughly interrogated by CMD simulations to find out their impact on the membrane function such as gas adsorption and separation.

### 2.1 Simulations of the Offset in Bilayer Nanoporous Graphene to Tune the Pore Size at sub-0.1 Å Resolution

**Figure 2:**
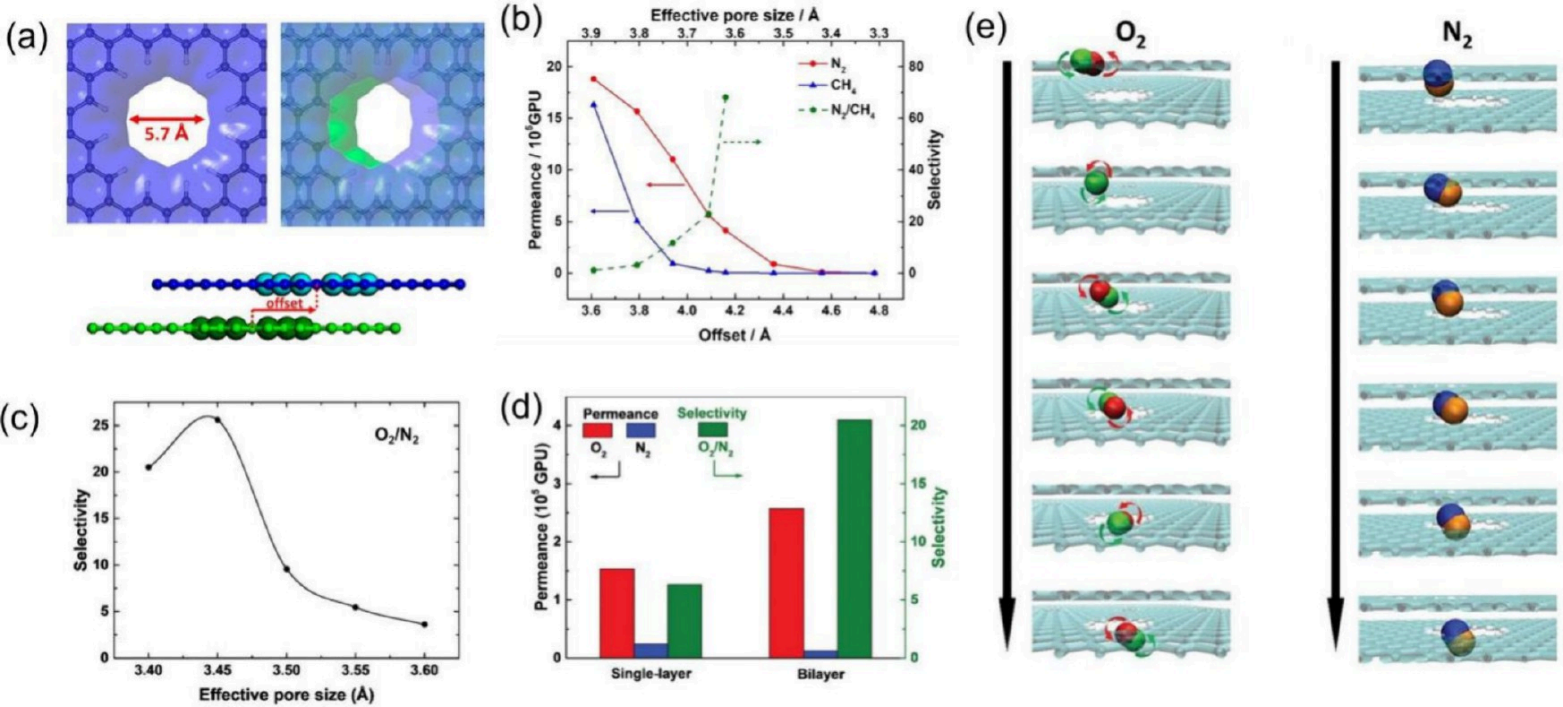
(a) Top and side views of bilayer nanoporous graphene membrane from stacking two porous graphene layers with an offset and (b) molecular dynamics simulations of N_2_/CH_4_ permeance and permselectivity vs the effective pore size and the offset of the bilayer graphene membrane. Adapted from ref (2). Copyright 2019 American Chemical Society. (c) Molecular dynamics simulations of O_2_/N_2_ permselectivity vs the effective pore size of the bilayer graphene membrane, (d) comparison of O_2_ and N_2_ permeance and selectivity for single-layer and bilayer graphene membranes with a similar pore size of 3.4 Å, and (e) snapshots of O_2_ and N_2_ passing through the bilayer nanoporous graphene membrane. Adapted from ref (29). Copyright 2019 Royal Society of Chemistry.

Since the advent of single layer graphene, numerous methods for creating pores^28^ have emerged. However, it is still challenging to precisely control the pore size in a one-atom-thin membrane for gas separation. To overcome these challenges in pore-size control for 2D membranes, a bilayer nanoporous graphene membrane (Figure 2a) was proposed with continuously tunable pore sizes.^2^ Varying the offset between the porous graphene layers allows for tunable effective pore sizes at 0.05 Å resolution, leading to high N_2_/CH_4_ selectivity (>60) at an effective pore size of 3.62 Å (Figure 2b) and an optimal O_2_/N_2_ selectivity at an effective pore size of 3.45 Å (Figure 2c).^29^ Interestingly, the optimal-offset bilayer configuration triples the O_2_/N_2_ selectivity of single-layer graphene with a similar pore size (Figure 2d). The enhanced selectivity arises from entropic effects —the slimmer and shorter O_2_ molecule rotates through the nanopore, while the longer and bulkier N_2_ molecule wiggles through with its rotation restricted (Figure 2e). The elliptic shape of the pore in the bilayer membrane (Figure 2a) is crucial in enabling the entropic selectivity, suggesting the importance of not only size control but also shape control of pores from stacking of 2D layers.

Simulation results in Figure 2 represent an ideal situation where one can precisely control the offset between two graphene layers and the relative positions of the two pores. The exact setup and control as shown in Figure 2a will be extremely challenging to achieve experimentally, because the potential energy surface of the bilayer as a function of the offset is relatively flat. However, the idea of pore alignment/misalignment is more general and has been recently employed experimentally to tune the pore mouth of 5A zeolites from 8 to 6 Å^27^ and the sizes of the aligned macrocycle pores in ultrathin films with angstrom precision by using different macrocycle molecules.^30^ The other general idea is to control stacking orders where more stable configurations such as AA vs AB can be obtained, as discussed below.

### 2.2 Experimental Realization of Stacking Control in COFs

**Figure 3:**
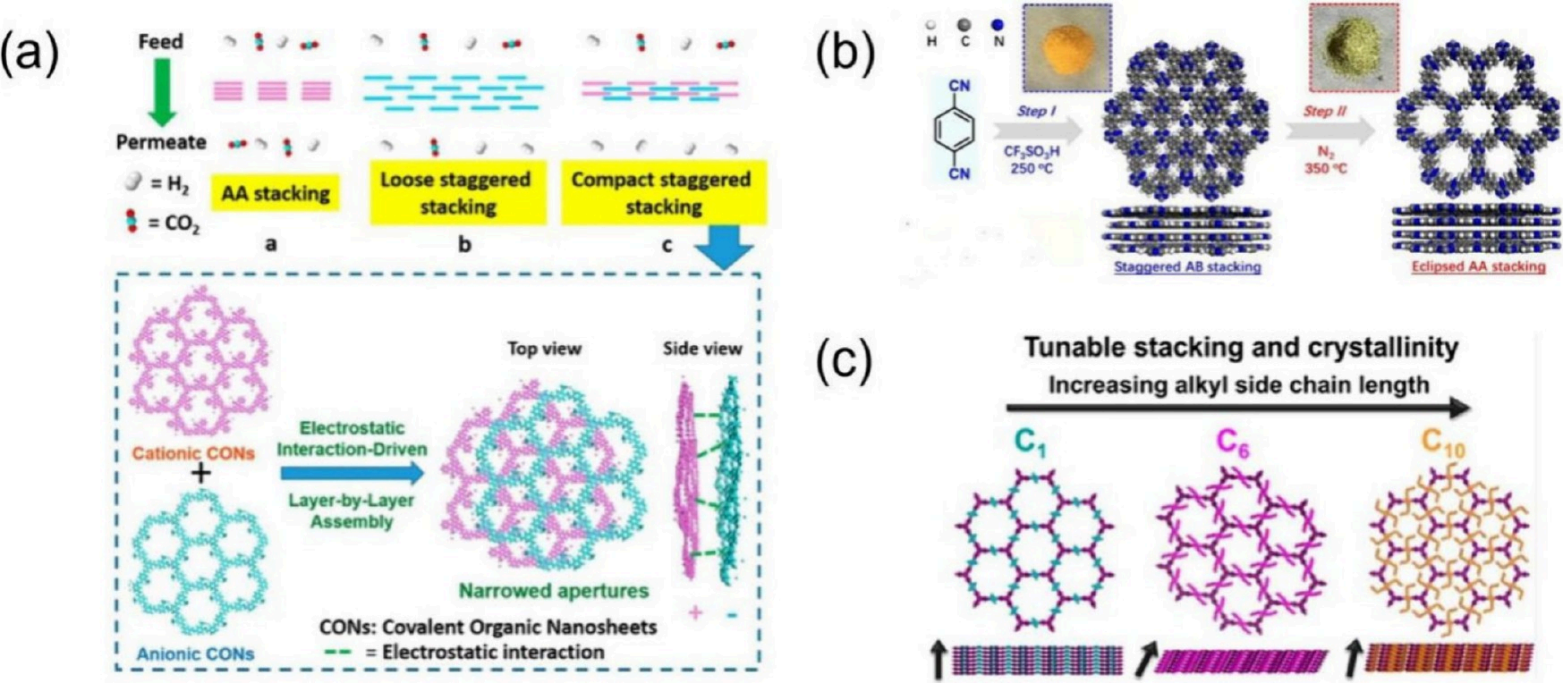
(a) Use of electrostatic attraction to stack covalent organic nanosheets (CONs). Adapted with permission from ref (31). Copyright 2020 American Chemical Society. (b) Use of thermal transformation to change from AB stacking to AA stacking. Adapted with permission from ref (32). Copyright 2020 American Chemical Society. (c) Use of side chains of various lengths to control COF stacking and crystallinity. Adapted with permission from ref (33). Copyright 2023 American Chemical Society.

COFs, known for their chemical tunability, high porosity, ordered molecular arrangements, and chemical stability, can be chemically processed to allow the manipulation of the individual porous 2D layers. Ying et al.^31^ used two intrinsically charged ionic covalent organic nanosheets with opposite charges to build ultrathin membranes with reduced apertures through electrostatic attraction (Figure 3a). This approach combines pore size mismatch, layer-by-layer assembly, and strong interlayer interactions to create compact ultrathin layers with staggered stacking and smaller pore sizes for selective H_2_ separation and transport. Yang et al.^32^ reported that the staggered AB stacking CTF-1 transforms into a highly crystalline CTF-1 with an eclipsed AA stacking mode under a nitrogen atmosphere at 350 °C (Figure 3b). This thermal transformation strategy can be extended to produce crystalline fluorinated CTFs with controllable fluorine content, high surface area, and tunable properties. Pelkowski et al.^33^ found that manipulating side chain interactions (via chain lengths, ranging from one to 11 carbon atoms) can effectively control the stacking and crystallinity of 2D imine-linked COFs (Figure 3c). Additionally, Ahmed et al.^34^ demonstrated that by altering the pH, layered COFs can undergo sliding and even separation between layers.

## 3 Ion-Gating

The ion-pore interaction in 2D materials offers additional control knobs to enrich the functions of 2D carbonaceous materials. Key design considerations include the cation-π interaction between the cations and the 2D layer, pore-anion interaction, the size difference between cations and anions, and electrostatic attraction between cations and anions. The fusion of these interactions led to the concept of ion gating whereby an anion hovers above the pore in a 2D layer and dynamically control the pore for gas permeation.

**Figure 4:**
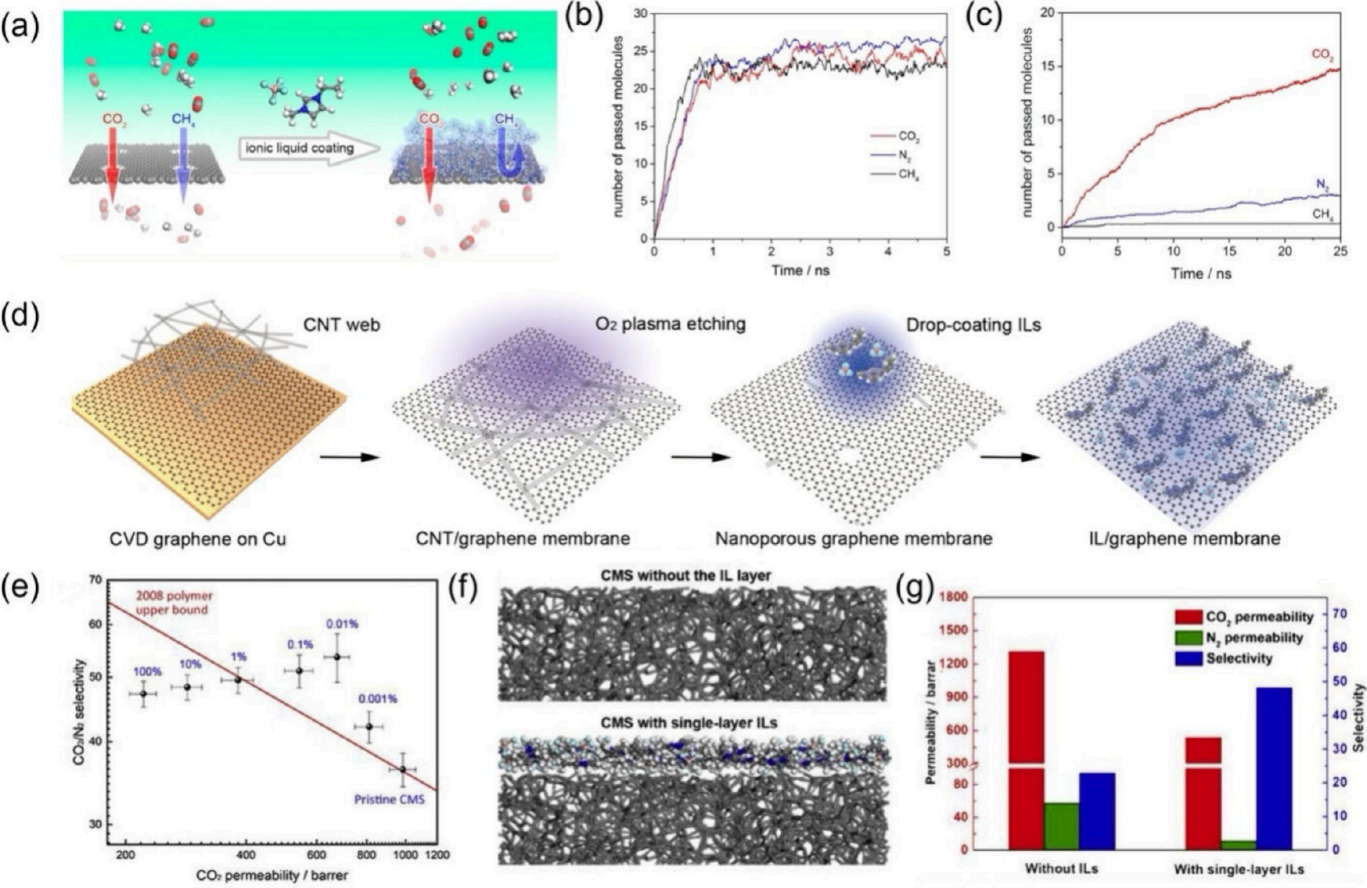
(a) Concept of ion-gating of porous graphene for selective gas permeation, (b) MD simulations of gas permeation through the uncoated porous graphene with a pore size of 5.7 Å, and (c) MD simulations of gas permeation through the [EMIM][BF_4_]-coated porous graphene with a pore size of 5.7 Å. Adapted with permission from ref (1). Copyright 2017 American Chemical Society. (d) Experimental synthesis of IL-coated porous graphene. Adapted with permission from ref (35). Copyright 2020 American Chemical Society. (e) Experimental performance of the CMS membrane coated with different mass loadings of the [BMIM][BF_4_] IL, (f) atomistic models of CMS membrane with and without a single-layer [BMIM][BF_4_], and (g) MD simulation results of gas permeation through CMS membrane with and without a single-layer [BMIM][BF_4_]. Adapted with permission from ref (36). Copyright 2020 Elsevier.

The design was based on a porous graphene membrane coated with an ionic liquid (Figure 4a).^1^ The pores in the graphene are 5.7 Å in size, so gases such as CO_2_, N_2_, and CH_4_ can all pass without any selectivity (Figure 4b). However, when a monolayer of [EMIM][BF_4_] ionic liquid (IL) is deposited on the porous graphene, MD simulations showed that selectivity appears as CO_2_ exhibits significantly higher permeance than the other two gases (Figure 4c). This is due to the anions dynamically adjusting the pore sizes by hovering above the pore and providing affinity to CO_2_, while the larger imidazolium cations hold the anions in place through electrostatic attraction. Inspired by the simulations, Guo et al. experimentally realized such a design by first growing graphene from chemical vapor deposition, then creating pores in the graphene sheet, and next drop-casting an ionic liquid layer (Figure 4d).^35^ Moreover, the ion-gating concept was demonstrated experimentally by using a carbon molecular sieve (CMS) membrane coated with an ultrathin IL layer as well (Figure 4e); MD simulations using atomistic models for the CMS and IL/CMS membranes (Figure 4f) confirmed the critical role of the IL layer in enhancing the selectivity (Figure 4g).^36^ Ion-gating is a promising strategy to enhance the selective transport capabilities in ultrathin carbon membranes, supported by both computational insights and experimental evidence. In addition, the concept of ion-gating has been applied in other materials as well, such as in zeolites^37^ and MOFs,^38^ indicating its broad applicability in tuning pore sizes and controlling molecular transport.

## 4 Interlayer Pillaring and Graphene/IL Interfaces

Different from ILs that act as a gate on porous graphene where the ion-pore interaction is the focal point, interlayer pillaring in 2D materials refers to the insertion of molecular or ionic pillars between the layers of 2D materials (which could be non-porous within the layers such as defect-free graphene sheets), to open up the interlayer space for various functions.

### 4.1 Simulations of IL-Pillared Graphene

**Figure 5:**
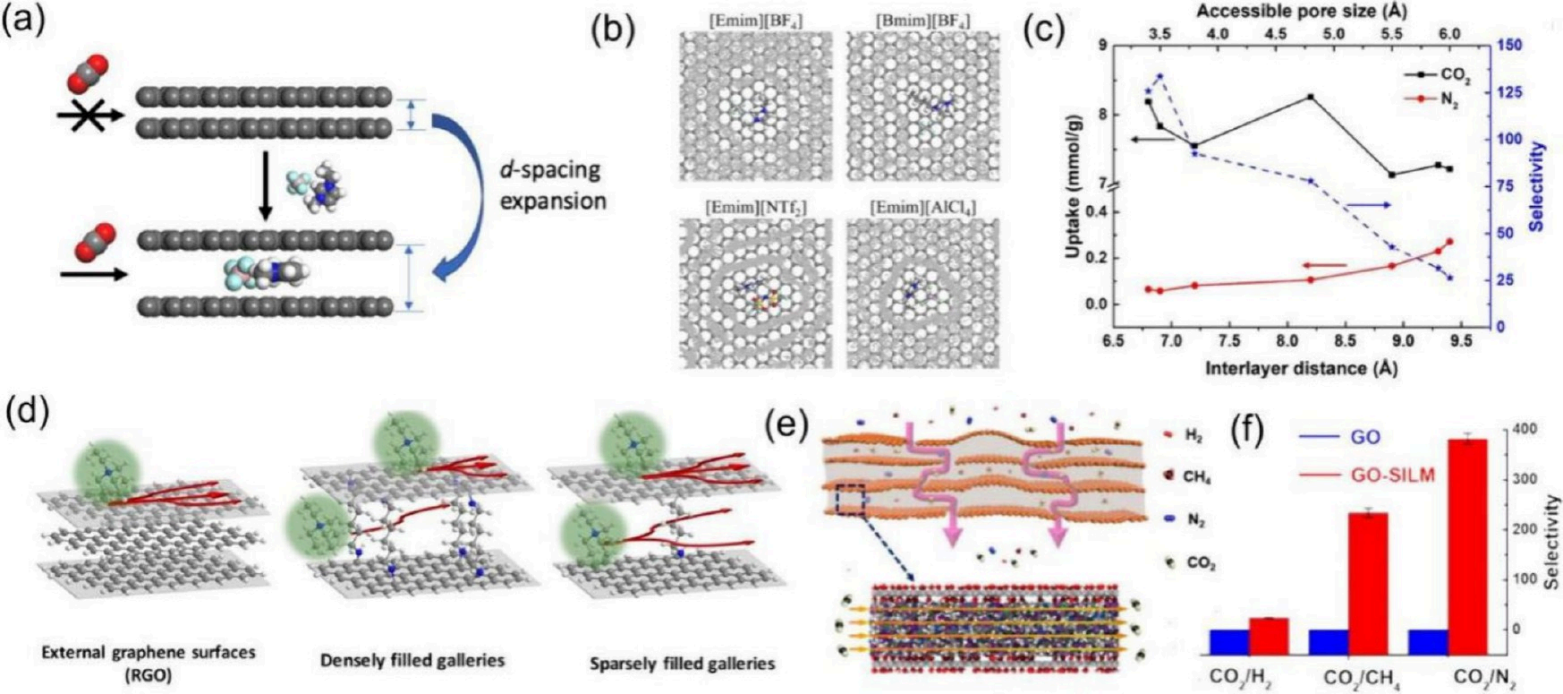
(a) Design of a graphene–ionic liquid (GIL) structure for gas adsorption with IL pillars, (b) top view of the distribution of CO_2_ binding sites (gray circles) in the interlayer space pillared by different ILs from GCMC simulations, and (c) GCMC-simulated gas uptakes and CO_2_/N_2_ selectivity in GIL structures of varying interlayer spacings and accessible pore sizes. Adapted with permission from ref (3). Copyright 2021 American Chemical Society. (d) Sparsely pillared graphene materials for fast ion transport and high-performance supercapacitors from reduced graphene oxide (RGO). Adapted with permission from ref (39). Copyright 2019 American Chemical Society. (e) Schematic illustration of the gas solution-diffusion transport pathway through a graphene-oxide-supported ionic liquid membrane (GO-SILM) and (f) CO_2_ separation performances of GO-SILM compared to GO. Adapted with permission from ref (40). Copyright 2018 American Chemical Society.

A graphene-ionic liquid (GIL) pillared structure was designed to utilize the slit pore between graphene layers.^3^Figure 5a illustrates the design concept: the interlayer distance in multilayer graphene, such as graphite, is only 3.4 Å, providing no accessible space for gas adsorption; by inserting ILs between the graphene layers, the interlayer space can be expanded to accommodate gas adsorption (Figure 5b). Different ionic liquids were selected to adjust the slit pore size, thereby optimizing gas uptake and selectivity. Density functional theory (DFT) calculations were used to optimize the GIL geometries and determine the slit pore sizes, and grand canonical Monte Carlo (GCMC) simulations were conducted to obtain gas-adsorption isotherms and selectivities (Figure 5c). The simulations revealed that high CO_2_/N_2_ and CO_2_/CH_4_ adsorption selectivities are achievable when the accessible pore size is less than 5 Å, highlighting the potential of GIL pillared structures for selective carbon capture. Interlayer pillaring combines the versatility of molecular chemistry with the layered nature of 2D materials and allows the interlayer space to be tunable and usable to enhance the properties and functionalities of 2D materials.

### 4.2 Synthesis of Pillared Graphene Systems

The simulated GIL systems in Figure 5a–c share many similarities with the well-known graphite intercalation compounds. Charged species such as Li-ions can be electrochemically intercalated between graphene layers. However, to intercalate both cations and anions at the same time or neutral pillars between graphene layers would need special consideration of the driving force. Banda et al. synthesized pillared graphene materials with varying interlayer separations and pillar density using alkyl diamines as pillars which are covalently attached to the graphene basal planes (Figure 5d)^39^ and found improved ion transport for high-performance supercapacitor applications. Ying et al. prepared a high-performance CO_2_-philic membrane by confining the [BMIM][BF_4_] ionic liquid within the nanochannels of a laminated GO membrane (Figure 5e).^40^ The confined ionic liquid serves as a fast transport channel for CO_2_, ensuring both high selectivity and high permeance (Figure 5f). The idea of interlayer pillaring goes beyond the graphene-based systems highlighted in Figure 5. For example, pillared structures of graphene by fullerenes^41^ and other 2D materials such as MXenes by amines^42^ have been synthesized.

## 5 Graphene Heterostructures

Not only can graphene interface with ionic liquids, but also with other 2D materials to form heterostructures. Here we can focus on the heterostructures of graphene with MXenes–2D carbides and nitrides with terminal surface groups, because the interfacial charge transfer can be tuned by the surface groups and the resulting local environment can be manipulated for ion transport and charge storage.

**Figure 6:**
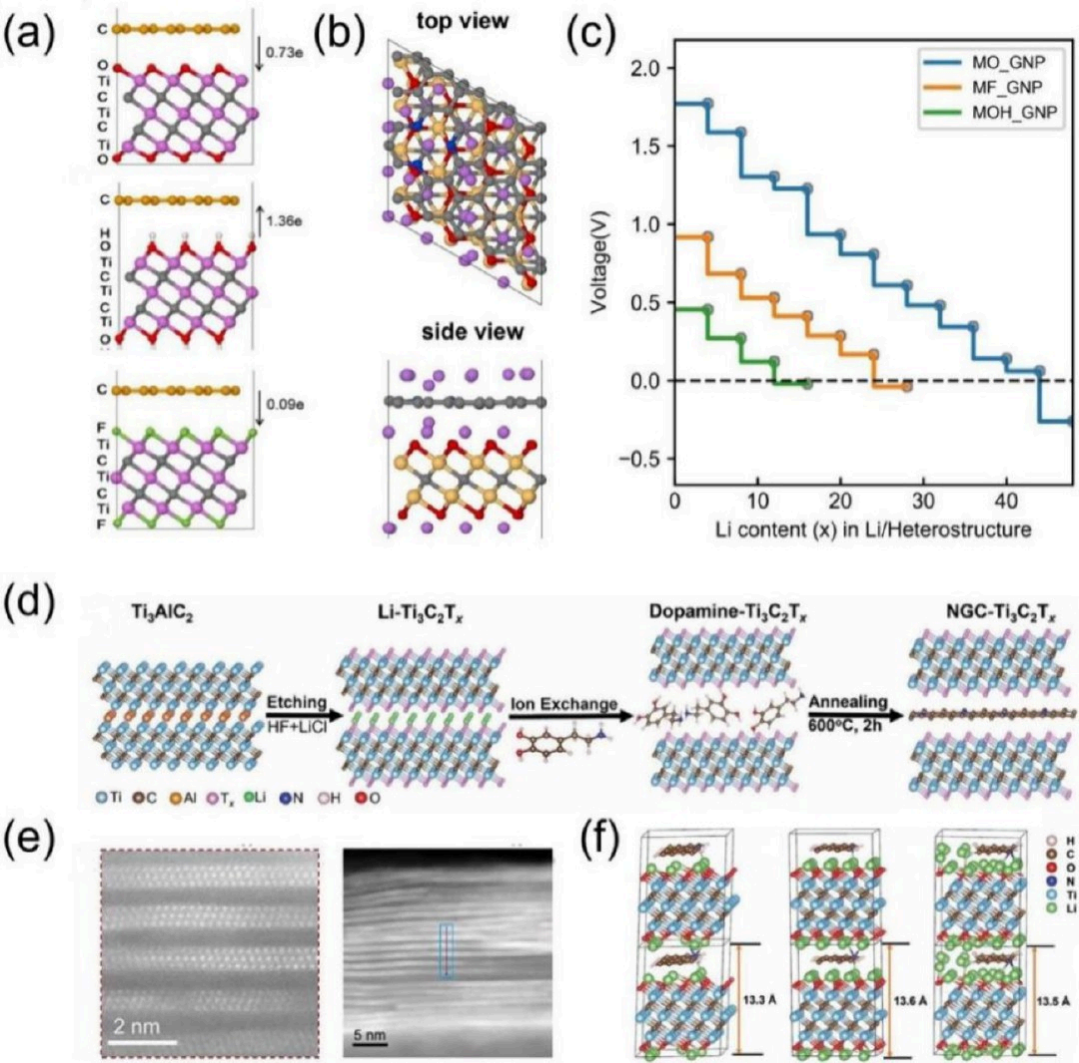
(a) Charge transfer between graphene and Ti_3_C_2_T_2_ MXenes with different termination groups (*T* = O, OH, F). Adapted with permission from ref (43). Copyright 2019 American Physical Society. (b) Adsorption configurations of Li in the heterostructure of Ti_3_C_2_O_2_ and graphene with N-doping of the pyridinic type (GNP) and (c) voltage profiles of Ti_3_C_2_O_2_/GNP (MO_GNP), Ti_3_C_2_F_2_/GNP (MF_GNP), and Ti_3_C_2_(OH)_2_/GNP (MOH_GNP) heterostructures with the Li insertion amount (in mol % per Ti_3_C_2_T_2_ unit). Adapted with permission from ref (44). Copyright 2020 Elsevier. (d) Schematic illustration of the preparation of the heterostructure of Ti_3_C_2_T_*x*_ with N-doped graphitic carbon (Ti_3_C_2_T_*x*_/NGC), (e) HAADF-STEM images of Ti_3_C_2_T_*x*_/NGC, and (f) optimized structures of Ti_3_C_2_O_2_/NGC with increasing Li insertion amounts. Adapted with permission from ref (45). Copyright 2024 Naguib et al.

The work function of a MXene is sensitive to the termination group; as a result, the charge transfer direction and amount between graphene and MXene can be tuned by varying the surface functional groups on the MXene surface. For example, DFT computation^43^ showed that electron transfers from graphene to the Ti_3_C_2_O_2_ MXene but from the Ti_3_C_2_(OH)_2_ MXene to graphene (Figure 6a). Further computation^44^ revealed that pyridinic N-doped graphene/Ti_3_C_2_O_2_ heterostructure (MO_GNP) has more capacity for storing Li^+^ ions (Figure 6b) than Ti_3_C_2_O_2_ alone. Moreover, the predicted voltage profiles (Figure 6c) indicate that the MO_GNP structure has higher charge capacity than the Ti_3_C_2_F_2_/GNP (MF_GNP) and Ti_3_C_2_(OH)_2_/GNP (MOH_GNP) heterostructures. Recently, Naguib and co-workers^45^ prepared nitrogen-doped graphene-like carbon/MXene heterostructure electrodes, by intercalating dopamine in the MXene layers and using it as a carbon precursor (Figure 6d). High-angle annular dark-field scanning transmission electron microscopy (HAADF-STEM) images of the Ti_3_C_2_T_*x*_/NGC material (Figure 6e) clearly show the MXene layers and the interlayer space where the nitrogen-doped graphene-like carbon (NGC) resides, confirmed by X-ray photoelectron spectroscopy. Li-ions were found to be stored at both the graphene/MXene interfaces (above and below the graphene layer) and the edge sites around the NGC (Figure 6f). The DFT-predicted capacity of 371 mAh g^–1^ agrees well with the experimental value of 400 mAh g^–1^.^45^

The charge transfer at the MXene/graphene interface also means that there is an electric field across the interface. The field and the surface groups on the MXene surface greatly influence the solvation environment for the ions confined between the MXene and the graphene layers. FPMD simulations of the proton/water confined in the interface showed that the O–H bonds could be oriented along the electric field, leading to a more oriented hydrogen-bond network and faster proton transport.^46^

## 6 2D Carbonaceous Materials beyond Graphene: 2D Fullerene Networks

Instead of using a sp^2^-carbon atom as the building block for a 2D material such as graphene, one can also use fullerenes as building blocks and link them covalently. In 1997, Oszlanyi et al.^47^ synthesized Na_4_C_60_, an alkali-intercalated 2D polymer, in a powder form. In 2002, Chen and Yamanaka reported the single crystals of a ’tetragonal’ C_60_ polymer under high pressure.^48^ In 2004, Margadonna et al. determined the structure of Li_4_C_60_ using powder XRD.^49^ In 2018, Tanaka and Yamanaka reported the ambient pressure vapor-phase growth of tetragonal Mg_2_C_60_ single crystals.^50^ The culmination of these developments came in 2022–2023 when Hou et al.^8^ and Meirzadeh et al.^51^ successfully synthesized single crystals of 2D covalent polymeric fullerene networks on gram scale under ambient pressures and found a new hexagonal phase (Mg_4_C_60_), expanding the 2D carbon family. More importantly, these single crystals like graphite can be exfoliated to single and few layers in thickness and the interstitial sites among the fullerene units provide a well-ordered array of angstrom-scale pores that can be employed for membrane separations.

### 6.1 2D Fullerene Networks

**Figure 7:**
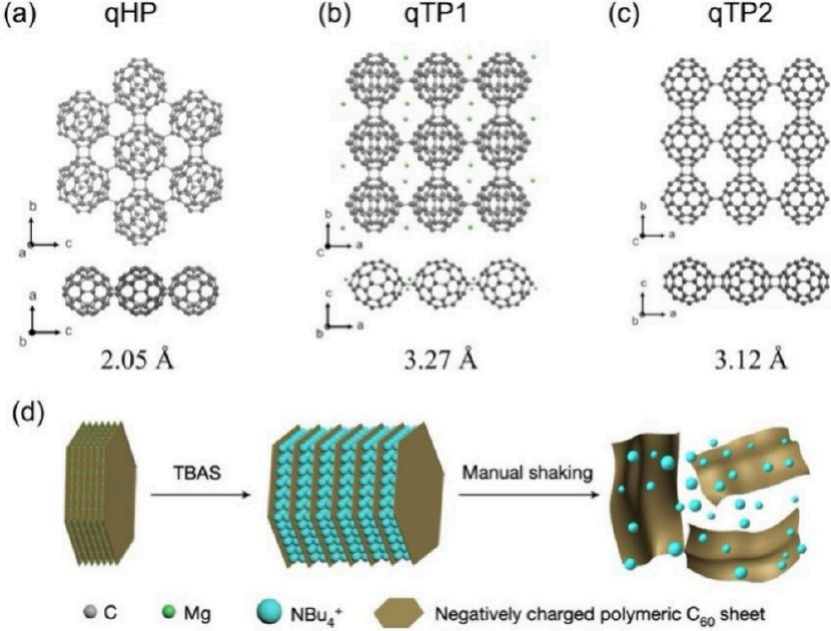
Top and side views as well as pore-limiting diameters of monolayer fullerene membranes from three different bulk phases: (a) quasi-hexagonal phase (qHP), (b) quasi-tetragonal phase 1 (qTP1), and (c) quasi-tetragonal phase 2 (qTP2). Adapted with permission from ref (4). Copyright 2023 American Chemical Society. (d) Organic cation slicing exfoliation: replacement of Mg ions (green) with larger NBu_4_^+^ ions (blue) in polymeric C_60_ layers, resulting in negatively charged nanosheets after gentle shaking. Adapted with permission from ref (8). Copyright 2022 Hou et al. under exclusive license to Springer Nature Limited.

There are three different monolayer fullerene membranes derived from different bulk synthesis processes: quasi-hexagonal phase (qHP, Figure 7a) with a pore-limiting diameter (PLD) of 2.1 Å, derived from Mg_4_C_60_; quasi-tetragonal phase 1 with a PLD of 3.27 Å (qTP1, Figure 7b), derived from Li_4_C_60_, Na_6_C_60_, and Mg_2_C_60_; quasi-tetragonal phase 2 with a PLD of 3.12 Å (qTP2, Figure 7c), derived from the high-pressure polymeric C_60_.^48^ Hou et al.^8^ used an organic cation slicing strategy to effectively exfoliate bulk single crystals into monolayer qHP flakes and few-layer qTP flakes (Figure 7d). Although the pores in the qHP 2D fullerene network are too small even for He, the pore sizes in qTP1 and qTP2 2D fullerene networks are in between H_2_ (2.9 Å) and other larger gases such as CO_2_ (3.3 Å) and O_2_ (3.46 Å) and can be used molecular sieving of H_2_ from other gases. The qTP1 membrane has one C–C single bond linking two fullerenes along the a-direction (Figure 7b bottom), while the qTP2 membrane has two parallel C–C single bonds (called [2 + 2] bonds) linking two fullerenes along the a-direction (Figure 7c bottom). It has been found that the C–C single bonds along the a-direction in the qTP1 membrane will break when the membrane is in the neutral state, leading to 1D polymerized fullerenes.^52^ In other words, the qTP1 membrane prefers to be in an anionic state and needs cations such as Mg^2+^ to be a stable 2D membrane.

### 6.2 Monolayer qTP Membranes for Hydrogen Separation

**Figure 8:**
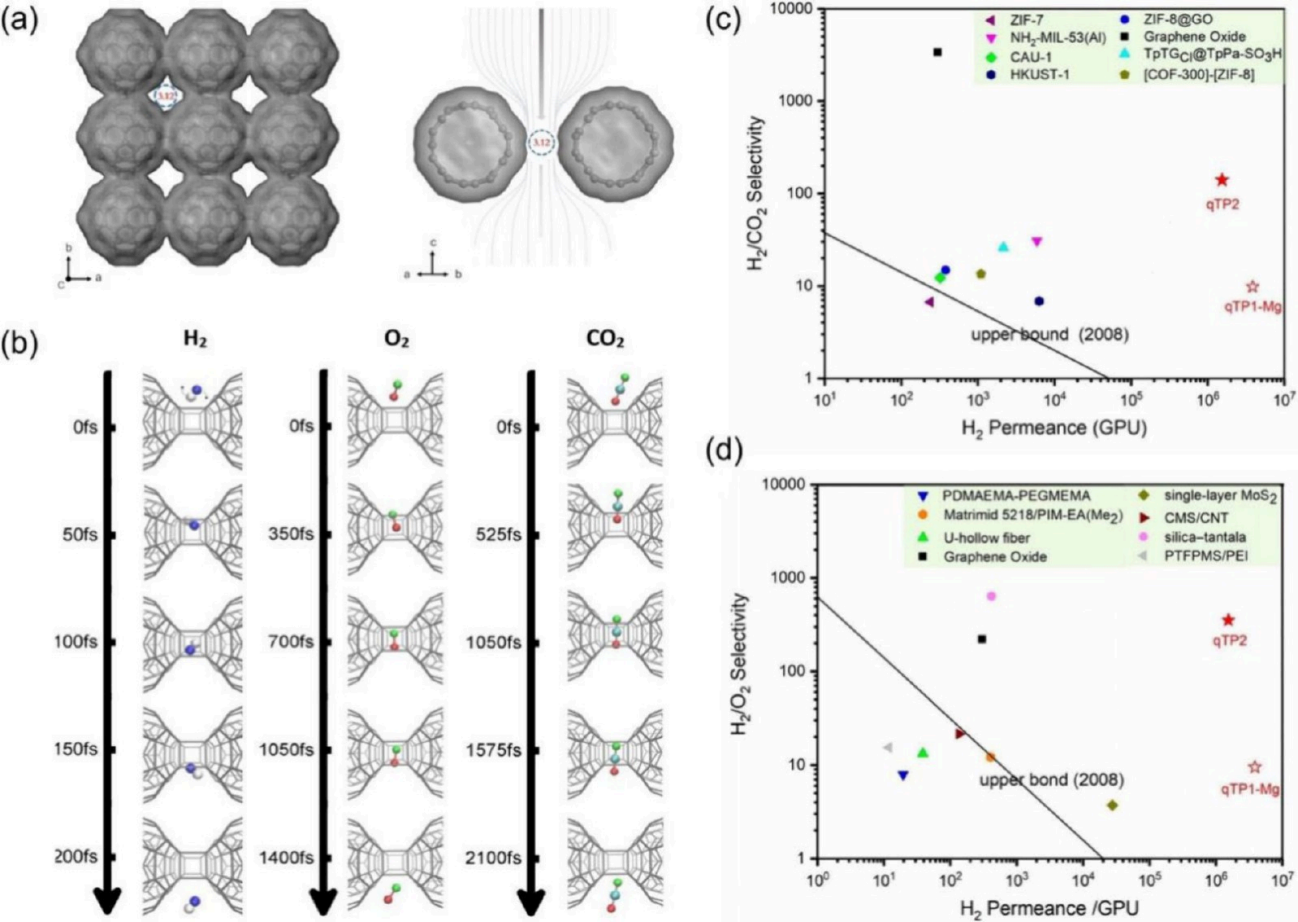
(a) Top (left) and side (right) views of the qTP2 membrane, showing 3D Connolly surfaces of funnel-shaped pores for gas transport. (b) Tracking a single permeation event across the qTP2 membrane for H_2_, O_2_, and CO_2_. Simulated selectivity vs permeance performances of qTP membranes for hydrogen separations in comparison with other materials: (c) H_2_/CO_2_ and (d) H_2_/O_2_. Adapted with permission from ref (4). Copyright 2023 American Chemical Society.

The qTP membranes have funnel-shaped pores, as seen from the side views in Figure 8a.^4^ The pore density is also notably high, at 1.2 per nm^2^, effectively increasing the active membrane area. Simulated pure-gas permeation across the qTP fullerene membranes using concentration-gradient-driven molecular dynamics (CGD-MD)^53^ shows that the two qTP membranes surpass the Robeson upper bounds for H_2_/CO_2_ and H_2_/O_2_ separations by a large margin (Figure 8c,d). The high H_2_ permeances are due to the qTP membranes’ high porosity and funnel-shaped pores. The lower H_2_ selectivity of the qTP1-Mg membrane (qTP-1 membrane stabilized by the Mg^2+^ ions) is attributed to its larger pore size. The higher H_2_ selectivity of the qTP2 membrane also stems from the entropic selectivity. As shown in Figure 8b, H_2_ rotates through the pore, while O_2_ and CO_2_ wiggle through the pore much more slowly; this entropic selectivity mechanism is similar to that of O_2_/N_2_ separation by the bilayer graphene membrane (Figure 2).

### 6.3 Porous qHP Membrane

**Figure 9:**
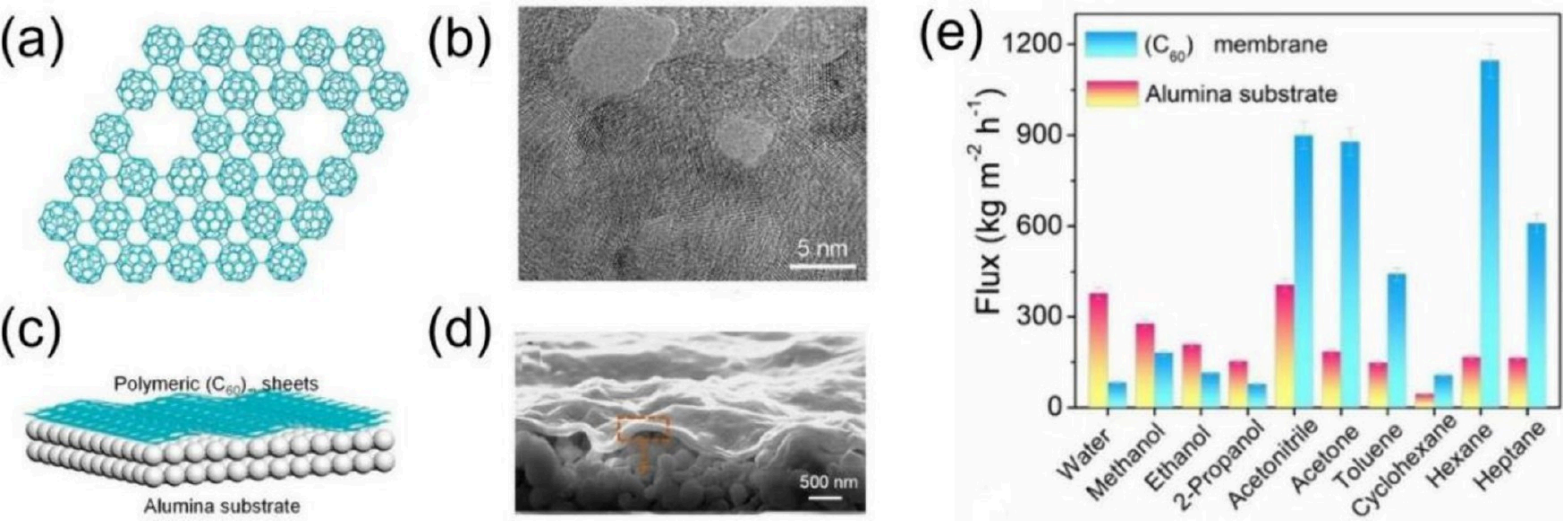
(a) Schematic illustration of porous qHP nanosheets in top view. (b) TEM image. (c) Schematic illustration of an alumina-supported porous qHP nanosheet membrane. (d) SEM image. (e) Measured performances of molecular separations by the qHP membrane vs the alumina substrate. Adapted with permission from ref (54). Copyright 2024 Wiley-VCH GmbH.

Although the pore size of the perfect qHP monolayer membrane (Figure 7a) is too small, Chen et al.^54^ created 1.2–5.3 nm nanopores in the plane of the qHP nanosheet (Figure 9a,b) by heating polymeric C_60_ crystals to 600 °C to achieve gentle depolymerization of covalently bonded C_60_ clusters. The ultrathin porous qHP membrane (Figure 9c,d) showed much faster transport of organic solvents than water (Figure 9e), demonstrating high separation performance and stability in various organic solvent nanofiltrations. This is because organic solvents pass through in vapor form, facilitated by the tailorable pores and lateral channels provided by the qHP nanosheets, while water passes through in liquid form.

## 7 Concluding Remarks and Outlook

We have provided an account of the latest advancements in the manipulation and computational design of 2D carbon-based materials in conjunction with the experimental verification and progress for molecular transport and functional interfaces. We highlighted the importance of precise atomic control over 2D structures to enhance properties like gas permeation, selectivity, membrane transport, and charge storage. Strategies such as controlled stacking of porous graphene bilayers, ion gating of pores in graphene, interlayer pillaring of graphene by ionic liquids, and heterostructure charge transfer between graphene and MXene were emphasized for their potential to improve functional properties. Despite the often-simplified model systems in the simulations, the concepts and strategies can often be translated to experimental systems. Conversely, the experimental progress in synthesizing 2D fullerene monolayers inspired simulations of their function for selective molecular separations to utilize their well-ordered subnanometer pores.

Looking ahead, creating new interactions and interfaces is key to discovering new functional 2D carbonaceous materials. For example, introducing metal atoms into the graphene layer can render it catalytic for reactions such as CO_2_ reduction,^55^ which can be combined with a CO_2_ selective membrane to preconcentrate the gas to accelerate the reduction reaction. On the other hand, great advances have been made in controlling the surface terminations in MXenes,^56,57^ which opens new avenues for heterostructures with graphene for charge storage. One also expects more efforts toward realization of the full potential of fullerene membranes in years ahead. In all these directions, molecular dynamics and density functional theory simulations, underscored in this account, will continue to play a critical role in guiding the design and understanding of new functional 2D carbonaceous materials.

Another potential fruitful area of exploration is to apply machine-learning (ML) in combination with simulations and experiments to advance the design of functional 2D carbonaceous materials. In a recent example,^58^ machine learning together with kinetic Monte Carlo and chemical graph theory was used to predict nanopore shapes and their formation probabilities and times in graphene; this work is very useful to understand the experimental kinetics of the top-down approach in creating pores in the graphene sheet and the resulting pore morphologies. In a latest example,^59^ the ML approach was integrated into a feedback loop in 2D membrane design and optimization which incorporates property prediction and data-driven insights using explainable artificial intelligence (AI). Using ML/AI to accelerate the generation of both fundamental insights and new designs and properties of 2D carbonaceous materials is exciting and we look forward to such successes in the near future.
